# Targeting Homeostatic T Cell Proliferation to Control Beta-Cell Autoimmunity

**DOI:** 10.1007/s11892-016-0731-9

**Published:** 2016-03-16

**Authors:** Debora Vignali, Paolo Monti

**Affiliations:** Transplant Immunology Unit, Diabetes Research Institute (DRI), IRCCS San Raffaele Scientific Institute, Via Olgettina 60, 20132 Milan, Italy

**Keywords:** Autoreactive T cells, Homeostatic proliferation, Interleukin-7, Type 1 diabetes, Islet transplantation

## Abstract

Immunomodulation of the autoreactive T cell response is considered a major strategy to control beta-cell autoimmunity, both in the natural history of type 1 diabetes and in islet transplantation, which can be affected by autoimmunity recurrence. So far, these strategies have had modest results, prompting efforts to define novel cellular and molecular targets to control autoreactive T cell expansion and activation. Novel findings highlighted the important role of the homeostatic cytokine interleukin-7 in inducing proliferation and differentiation of autoreactive T cell clones that causes beta-cell autoimmunity. In this review, we discuss recent evidences and novel findings on the role of IL-7 mediated homeostatic T cell proliferation in the process of beta-cell destruction and evidences of how targeting IL-7 and its receptor could be an innovative and effective strategy to control beta-cell autoimmunity.

## Introduction

Type 1 diabetes is caused by the selective destruction of pancreatic insulin-producing beta-cells by an immune-mediated reaction, predominantly mediated by autoreactive T cells [1]. CD4+ and CD8+ T cells able to recognize MHC class II and class I restricted epitopes of the beta-cell-associated antigens glutamic acid decarboxilase 65 (GAD65), proinsulin, islet tyrosine phosphatase (IA-2), and zinc transporter 8 [2] have been found in patients with type 1 diabetes using proliferation assays, ELISPOT, and fluorescent class I and class II MHC multimers [3]. These studies have also highlighted several key observations concerning the nature of the T cell response toward beta-cells. First, while naive T cells specific for beta-cell antigens are commonly found in subjects with no signs of beta-cell autoimmunity [4•], in patients with type 1 diabetes autoreactive T cells show signs of previous antigen encounter, such as telomere shortening [5•], activation in the absence of co-stimulatory signals [6], and the expression of the memory marker CD45RO [5•]. Second, an autoreactive memory T cell response is difficult to control with standard immunosuppression and is highly refractory to immunomodulatory molecules. This is testified by the limited efficacy of recent clinical trials aiming to prevent or delay immune-mediated beta-cell loss using GAD65 vaccination [7], CTLA4-Ig [8], and humanized anti-CD3 antibody [9]. More aggressive approaches based on profound T cell depletion, although effective early after treatment [10], were later affected by frequent relapse of the autoimmune response [11, 12]. Third, generation and expansion of autoreactive T cell clones can occur under the influence of homeostatic proliferation mediated by interleukin-7 (IL-7) [13]. The canonical antigen-specific proliferation pathway relies on the autocrine production of IL-2 and the upregulation of the IL-2 receptor alpha (also known as CD25). Therefore an important class of immunomodulatory molecules was developed to target this pathway including calcineurin inhibitors and anti-CD25 antibodies. Recent evidences however clearly showed that T cells can proliferate and acquire a memory phenotype upon activation of the IL-7/IL-7R axis [14••]. Moreover, recent findings suggest that IL-7 is involved in the generation of T cells with a stem cell-like memory phenotype (memory stem T cells, Tscm) [15••] and in the reprogramming of T cell bio-energetic metabolism for T cell proliferation [16]. While the interest on the homeostatic T cell proliferation pathway is increasing in autoimmunity, there is a substantial lack of molecules targeting this pathway in humans and trials to assess whether this could be an effective approach to control T cell-mediated beta-cell autoimmunity. In this article, we discuss how the IL-7/IL-7R pathway can contribute to the development of type 1 diabetes and how preclinical models have demonstrated the efficacy of a selective targeting of this pathway. Finally, we discuss how, and in which clinical setting, the targeting of the IL-7/IL-7R pathway can be a therapeutic option for the prevention and treatment of beta-cell autoimmunity.

## IL-7 Production and Regulation

**Fig. 1:**
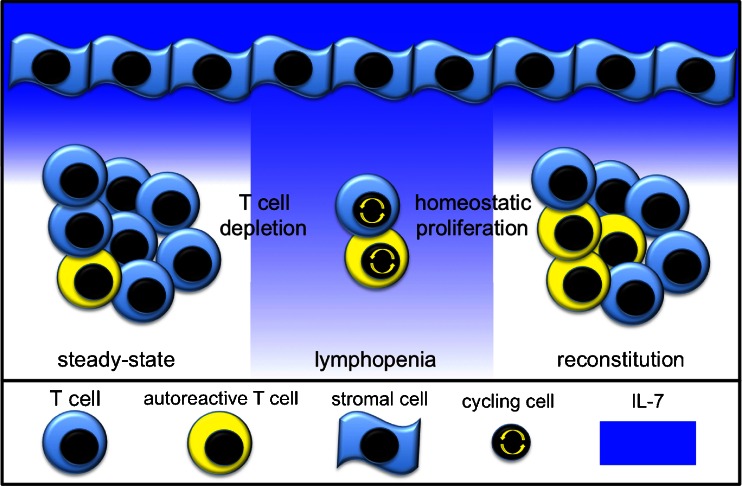
IL-7-mediated homeostatic T cell proliferation. In normal conditions, stromal cells constitutively produce IL-7 which is consumed by T cells and kept at low levels in the periphery. After T cell depletion, IL-7 concentration becomes elevated and remaining T cell start to proliferate in the IL-7 rich milieu. During expansion, autoreactive T cell clones can undergo expansion and are included in a higher number in the reconstituted T cell pool

IL-7 is secreted by stromal cells located in the bone marrow, in peripheral lymphoid organs, and in the gastro-intestinal tract [17]. These cells have yet to be fully characterized; however, it appears that IL-7 transcripts are produced at a constitutive level and are not influenced by extrinsic stimuli such as the concentration of IL-7 in a negative feedback or the size of the T cell compartment. Peripheral IL-7 concentration relies on consumption by IL-7 receptor positive T cells. IL-7 production at a fixed rate and consumption keeps the serum concentration of IL-7 below 5 pg/ml in physiological conditions. [18] This represents a limiting factor for T cell expansion. In an immune system with a full T cell compartment, the concentration of IL-7 is sufficient for the survival of a finite number of T cells. IL-7 also maintains a slow rate of T cell proliferation to counterbalance physiological T cell death. When the peripheral T cell number is reduced (lymphopenia), the serum concentration of IL-7 rises to supraphysiological levels (Fig. 1). Remaining T cells in a lymphopenic environment and in an IL-7 rich milieu enter the cell cycle, and homeostatic T cell proliferation induced by IL-7 occurs. The process is self-limiting as restoring the number of peripheral T cells corresponds to an increased consumption of IL-7. Homeostatic T cell proliferation stops when the physiological T cell number and IL-7 concentration are fully restored.

## The IL-7/IL-7 Receptor Signaling Pathway

The high affinity IL-7 receptor results from the IL-7-mediated crosslinking of the extracellular domains of the IL-7 receptor alpha-chain (IL-7Rα; also known as CD127) and the common cytokine receptor-γ chain (γ_c_, also known as CD132). Tyrosine kinases Janus kinases (JAK1) and JAK3 which are linked to the intracellular domain of the γ_c_ mutually phosphorylate and their increased kinase activity, thus inducing phosphorylation of the IL-7Rα intracellular domain and allowing the binding of the signal inducer transducer and activator of transcription factor 5 (STAT-5) which dimerizes and translocates to the nucleus activating gene transcription [19]. Once in the nucleus, STAT5 regulates the expression of many targets genes involved in T cell proliferation and survival, including the upregulation of prosurvival Bcl-2 family members, such as the main targets BCL-2, MCL-1, and BC-XL [20] and downregulation of proapoptotic members of the BCL-2 family, such as BAX or BIM [21]. Other STAT5-regulated genes are Runt-related transcription factor 3 (Runx3), essential for T cell development and differentiation, and the suppressor of cytokines signaling (SOCS), necessary for negative feedback regulation of the cytokine signaling and T cell compartment homeostasis.

In addition to survival, the IL-7R signaling induces T cell proliferation through the repression of the cell cycle inhibitor p27^kip1^ [22] and preventing degradation of Cdc25A [23]. T cell proliferation is an energy-demanding process and necessitates the incorporation of nutrient to increase the biomass needed to produce daughter cells [24]. In order to meet the bio-energetic demands for proliferation, T cells activate aerobic glycolysis. Despite a lower efficiency in ATP production as compared to oxidative phosphorylation, glycolysis is an anabolic process and provides also fundamental nutrients for the synthesis of biological macromolecules [25]. The IL-7 signaling activates the PI3K/Akt pathway, which is fundamental for T cell proliferation. To sustain the bio-energetic and metabolic needs, IL-7R signaling is also involved in the activation of the glycolytic machinery, including the Akt-mediated upregulation of the glucose transporter 1 (GLUT1) [26] on the cell surface and the upregulation of the glycolytic enzymes hexokinase II (HXKII) [16]. Excision of IL-7R on mature T cells in vivo results in size and growth reduction of T cell compartment due to homeostatic mechanism impairment and metabolic cellular machinery liability [27].

## IL-7 in Beta-Cell Autoimmunity

In order to protect an individual from a wide range of different pathogens, the T cell compartment needs to guarantee a broad diversity of antigen specificity and a sufficient cell number to mount an effective immune response [28]. The T cell repertoire can also include T cell clones reactive to self-antigens, which escaped the negative selection process in the thymus and circulate as naive T cells. These potentially pathogenic clones are prevented from activation and proliferation mainly by their low affinity for the cognate MHC-peptide antigen complex which can deliver only weak signals as compared to high affinity T cell receptors [29•]. The TCR signal strength determines the T cell capacity to produce IL-2 and to upregulate the IL-2Rα (also known as CD25) for an effective expansion and differentiation in a T cell compartment with a self-limiting size [30].

This competitive advantage of T cells with a high affinity TCR is lost in an IL-7 rich milieu. While the IL-2Rα has an inducible expression regulated by T cell activation through the TCR, the IL-7Rα is constitutively expressed by all T cells and can induce T cell proliferation when the circulating concentrations of IL-7 reach supra-physiological levels [28]. Moreover, the low affinity TCR-MHC self-peptide interactions, which are required for T cell persistence, can give a selective advantage to autoreactive T cell clones [29•]. Perturbations of the IL-7/IL-7R axis can be associated with the activation of autoreactive T cells.

In animal models, the non-obese diabetic (NOD) mouse was shown to be affected by a chronic state of lymphopenia, which is permissive for autoreactive homeostatic T cell expansion under the influence of IL-21 [31]. Increasing the number of circulating T cells by injection of complete Freund adjuvant (CFA) at 3 weeks of age efficiently reduced the incidence of diabetes. Direct evidences of the role of IL-7 in the autoimmune beta-cell destruction process come from the rat insulin promoter (RIP)-lymphocytic choriomeningitis virus glycoprotein (GP)-transgenic mouse model [32•]. In this model, adoptive transfer of Smarta T cells restricted for the I-Ab-restricted LCMV GP61-80 (p13) epitope together with polyclonal CD8 T cells did not modify the incidence of diabetes. An increase in the incidence of diabetes was reported only when lymphopenia was induced with cyclophosphamide or by exogenous administration of IL-7. The diabetogenic effect of cyclophosphamide or IL-7 injection can be neutralized by blocking the IL-7R with a monoclonal antibody.

Lymphopenia, which is associated with increased circulating levels of IL-7 represents a typical condition in which autoreactive T cells can expand [28]. A relatively high risk of autoimmunity development has been observed in patients undergoing bone marrow transplantation after conditioning regimen and profound T cell depletion. Immunosuppression in patients receiving islet allotransplantation was associated with IL-7-mediated expansion of autoreactive T cell clones specific for GAD65 [14••]. Patients at risk for or with diagnosed type 1 diabetes have normal circulating levels of IL-7. However, single nucleotide polymorphisms (SNPs) of the IL-7Rα were associated with an increased risk of developing type 1 diabetes and multiple sclerosis [33]. As for many other cytokine receptors, a soluble form of the IL-7Rα has been identified and characterized for its capacity to bind to and inhibit the interaction of IL-7 with the surface receptor in T cells. In conditions of high blood glucose levels, the soluble IL-7Rα undergoes a non-enzymatic glycation process, losing its buffering capacity of IL-7 biological activity [34]. High levels of circulating IL-7 can be found in neonates and persist for the first 6 months of life [35]. Both GAD65 and proinsulin-specific T cells with a naive phenotype are already present at birth, and IL-7 was shown to promote in vitro proliferation and activation of GAD65 and proinsulin-specific T cells obtained from cord blood samples.

An IL-7 rich milieu can affect immune tolerance to self-antigens by acting directly on the CD4+CD25+ regulatory T cells (Treg) [36•]. The Treg network is essential to limit proliferation of autoreactive T cell clones. Despite a low expression of the IL-7Rα, which makes Treg unresponsive to physiological concentrations of circulating IL-7, STAT5 phosphorylation can be detected at pathological circulating concentrations of IL-7. The Treg response to IL-7 appeared to correlate with the phenotype. While CD45RO+ memory Treg remain anergic, CD45RO naive Treg strongly proliferate in response to IL-7, thus acquiring a CD45RO+ memory phenotype. Importantly, once exposed to IL-7, both memory and naive Treg have a reduced capacity to inhibit proliferation of conventional T cells in response to autoantigens. The suppressive capacity is fully restored upon IL-7 withdrawal. This effect can be of importance in the islet transplantation setting in which prolonged exposure of Treg to high circulating concentrations of IL-7 can impair the Treg capacity to control the expansion of autoreactive and alloreactive T cells.

## IL-7 and the Generation of Memory Stem T Cells

The memory T cell compartment is composed by several subsets in different stages of differentiation [30]. Conventionally, memory T cell populations can be divided according to the differential expression of CD45RA, CCR7, and CD62L into central memory (Tcm), effector-memory (Tem), and terminally differentiated effector-memory RA (Temra) [37]. All memory subsets are generated from a common naive T cell (Tn) precursor according to a progressive differentiation model [38]. In this model, Tn progress along a differentiation pathway in the order of Tn, Tcm, Tem, and Temra. The final step of differentiation generates terminally differentiated effector T cells that are short lived and undergo to massive apoptosis (contraction) when the immune response exhausts. It was unclear whether long-term T cell memory is maintained by the conventional memory T cell subsets or by a long-lived T cell precursor with self-renewal potential. In humans, conventional memory T cell subsets have indeed a rapid turnover and a half-life of few weeks [39, 40]. The existence of a memory precursor with stem cell-like properties was first hypothesized and subsequently described in mice [41••], in humans [42••], and in non-human primates [42••]. Tscm show a CD45RA+CCR7+ naive phenotype associated with the expression of the memory markers IL-2Rbeta and CD95 [42••]. Generation of Tscm from naive T cells involves the homeostatic cytokine IL-7. In the classical antigen-specific activation pathway, T cell receptor engagement and IL-2 provides strong signals for T cell differentiation toward short-lived effector cells. In contrast, the homeostatic cytokine IL-7 sustains T cell proliferation without the robust differentiating activity of IL-2. In vitro priming of naive T cells in the presence of IL-7 results in the generation of T cells with phenotypic, functional, and gene expression attributes found in naturally arising Tscm cells [15••].

This is relevant to beta-cell autoimmunity because T cell memory to beta-cell antigens is established before the onset of type 1 diabetes and is then maintained for decades after the disease onset, as testified in patients with type 1 diabetes receiving islet or pancreas transplants, in which despite immunosuppression, can be associated with reactivation of autoimmunity [43, 44]. Given the role of the IL-7/IL-7R axis in T cell autoimmunity and the long lasting memory to beta-cell antigens observed in patients with type 1 diabetes, we previously hypothesized that a T cell precursor with stem cell-like properties could be generated by autoantigen stimulation in the presence of IL-7 [45]. Even though the existence of memory stem T cells reactive to beta-cell antigens has to be proven, this population would be an ideal target cell for an innovative approach to beta-cell autoimmunity. Most strategies were designed to target activation/proliferation of pathogenic effector T cells. However, the identification and targeting of T cells that preserve long-term memory to beta-cell antigen would be able to eradicate the pathogenic T cell progeny. This approach is similar in principle to that hypothesized to treat cancer by targeting cancer stem cells [46]. In this theory, a permanent eradication of a tumor mass can be achieved by selective targeting of rare and slowly proliferating cancer stem cell precursors. In contrast, standard radio-chemotherapy targeting the vast majority of rapidly proliferating cancer cells but not cancer stem cells results in a high rate of tumor relapse.

## Targeting IL-7 Mediated Homeostatic T Cell Proliferation in Preclinical Models

Molecules with the ability to directly or indirectly target the IL-7/IL-7R axis have been discovered and first tested in preclinical models (Table 1). The IL-7/IL-7R axis has been implicated in the development of autoimmune diabetes in the NOD mouse model. Two reports showed how IL-7Rα blockade was effective to prevent and revert diabetes. In the first report [47••], the selective inhibition of the IL-7Rα using a monoclonal antibody was able to prevent diabetes after only 2–3 injections starting at week 9 of age. Disease remission was complete and durable, after a 4-week cycle of anti-IL-7Rα antibody starting at the time of diabetes onset. Anti-IL-7R antibody was reported to act by inhibiting diabetogenic effector-memory T cells (Tem). Diabetogenic Tem cells were not depleted by IL-7Rα treatment but were instead reprogrammed to suppress IFN-γ secretion and to upregulate the inhibitory receptor programmed death 1 (PD-1). Tem cells from anti-IL-7Rα-treated mice were also adoptively transferred in recipient mice without causing diabetes and demonstrating that IL-7-signaling deprivation was related to a state of cell intrinsic tolerance. In the second report [48••], treatment with the IL-7Rα antibody resulted in the reduction of IFN-γ-producing CD4+ (TH1) and IFN-γ-producing CD8+ (Tc1) T cells. As shown also in the first report, IL-7Rα antibody treatment induced upregulation of PD-1 expression in effector T cells that appeared to be necessary for maintenance of tolerance, which could be reversed by PD-1 blockade. Interestingly, in both models, IL-7Rα antibody treatment was associated with an increased frequency of regulatory T cells. The same monoclonal antibody raised against the IL-7Rα was also tested on BALB/c mice receiving C57BL/6 islets under the kidney capsule, as model of islet transplantation in streptozotocin-induced diabetes [49•]. When started 3 weeks before islet infusion, the anti-IL-7Rα treatment induced indefinite pancreatic islet allograft survival. This is of interest in the field of islet transplantation where a single treatment with an anti-IL-7Rα can potentially control both the autoimmune and the alloimmune response to islet allografts.

**Table 1:** Molecules targeting the IL-7/IL-7R axis

Study phase	Molecule	IL-7/IL-7R specificity	Setting	Refs
Clinical	Anti-IL-7Rα mAb	Yes	T1D, MS	N/A
	Mycophenolate mofetil	No	Islet tx in T1D	[14••]
	JAK inhibitors	No	RA	[55]
Preclinical	Anti-IL-7Rα mAb	Yes	Diabetes in NOD Islet tx	[47••, 48••, 49•]
	Bz-423	No	GVHD	[59•]
	2-DG+ metformin	No	Lupus	[60•]
Experimental	sCD127	Yes	Human	[34]
	Tat protein	Yes	Human	[54]

Biological and pharmacological regulators of the IL-7/IL-7R axis with a potential translation into the clinic have been identified. As for other cytokine receptors, a soluble form of the IL-7Rα (sCD127) has been identified in humans [50]. sCD127 is generated both by alternative splicing or shedding of the membrane bound IL-7Rα. The amount of circulating sCD127 is regulated by single nucleotide polymorphism (SNP) of the IL-7Rα gene [51]. A SNP found in exon 6 (rs6897932) determines the extent of exon splicing. Transcripts that skip exon 6 (C allele of rs6897932) encode sCD127 [52]. The polymorphism determines an increase of circulating levels of sCD127 and has been associated with an increased susceptibility to type 1 diabetes. sCD127 can bind to and inhibit IL-7 and is the only known endogenous regulator of the IL-7 biological activity [34]. sCD127 display a low affinity (Kd = 10–8 M) for IL-7 which is 3 logs lower than the affinity of IL-7 for the membrane IL-7Rα-γ_c_-chain [53]. Nevertheless, sCD127 concentration in serum (70–80 ng/mL) is 4 logs higher than physiological IL-7 concentration IL-7 (5 pg/ml) and is therefore expected to significantly influence the IL-7 bioactivity. The generation of a modified sCD127 with higher affinity for IL-7 could have therapeutic applications.

An interesting biological compound, which acts as a potent downregulator of IL-7Rα signaling is the 15 kDa Tat protein produced by the HIV virus [54]. Tat is secreted by HIV infected cells detectable in the serum of HIV-infected patients or in supernatants from in vitro cultures. Tat enters the cytoplasm of CD8+ T cells and interacts with the cytoplasmic tail of the IL-7Rα causing clustering and downregulation from the cell surface. The internalized IL-7Rα is then directed to the proteasome for degradation.

Pharmacological modulation of IL-7Rα signaling can be achieved by targeting downstream signaling such as JAK1 and JAK3. Tofacitinib is a potent inhibitor of JAK1 and JAK3 and currently approved for the treatment of rheumatoid arthritis (RA), has demonstrated effectiveness in the treatment of psoriasis in phase III clinical trials, and is currently tested as immunomodulator for organ transplantation [55]. Tofacitinib was shown to impair Th1 and Th17 effector function; however, its action is broad and includes all γ_c_-chain cytokines. Other JAK inhibitors under evaluation in clinical trials for autoimmune diseases include the JAK1 and JAK2 inhibitor Ruxolitinib, the JAK1 inhibitor GLPG-0634, and the JAK3 antagonist VX-509. Even though none of these compounds is selective for the IL-7/IL-7Rα signaling pathway, they can be used in combination with selective inhibitors of the IL-7Rα to potentiate the effectiveness.

## Targeting IL-7-Mediated Homeostatic T Cell Proliferation in Type 1 Diabetes and Islet Transplantation

A humanized IgG1 monoclonal antibody that binds to and inhibit the IL-7Rα has been developed by GlaxoSmithKline and tested as single ascending doses study phase I clinical trial on healthy volunteers to determine the safety, tolerability, pharmacokinetics, pharmacodynamics, and immunogenicity (NCT02293161, ClinicalTrials.gov identifier). The primary outcome measures were expected in September 2015 and not yet released.

The anti-IL-7Rα monoclonal antibody PF-06342674 (RN168) developed from Pfizer has been tested in phase 1b in patients with multiple sclerosis (NCT02045732 ClinicalTrials.gov identifier) with the primary outcome to evaluate adverse side effects and pharmacokinetic–pharmacodynamic properties. The trial is reported to have been terminated in April 2015 due to a corporate decision not related to safety or tolerability issues. A second trial was designed to test the same antibody in patients with type 1 diabetes and is estimated to conclude in March 2016.

While waiting for the results of these trials in terms of safety and tolerability and for the design of phase II and phase III trials to determine whether blockade of the IL-7Rα is effective in the treatment of type 1 diabetes, it is important to highlight the interest and the effort to explore the IL-7/IL-7R axis as a potential target pathway to control beta-cell autoimmunity.

As we previously reported, islet transplantation in patients with type 1 diabetes is associated with IL-7-mediated homeostatic proliferation of memory autoreactive T cells. This model also provided important evidences of the effect of classical immunosuppressive drugs on the IL-7/IL-7R axis. The mTOR inhibitor rapamycin and the calcineurin inhibitor FK506 were ineffective on IL-7-mediated homeostatic T cell proliferation at therapeutic serum level concentrations [14••]. Interestingly, the anti-CD25 antibody daclizumab by blocking the interaction of CD25 with the γ_c_ chain, promotes the association of the IL-7Rα/γ_c_ complex resulting in an increased sensitivity of T cells to IL-7 [56]. On the other hand, mycophenolate mofetil (MMF) was shown to reduce by 90 % the rate of homeostatic T cell proliferation at therapeutic serum level concentrations [14••]. MMF is a purine synthesis inhibitor acting very downstream in the proliferation process. Even though the mechanisms of action suggest that inhibition of T cell proliferation is not specific it represents the best therapeutic option to control homeostatic proliferation at least in the transplantation setting.

## Future Perspectives

Recent findings suggest that the immune response can be manipulated by targeting the bio-energetic metabolism of T cells. Both naive and memory T cell subsets are quiescent cells and rely on oxidative phosphorylation to sustain housekeeping functions [57]. However, proliferating cells like activated and effector T cells upregulate oxidative phosphorylation to meet the increased energetic demand and activates aerobic glycolysis, which is better suited for the biosynthesis of cell components, proliferation, and effector functions [58]. Bz-423 is a novel small molecule that inhibits the mitochondrial F1F0-ATPase resulting in the increase of superoxide and inducing selective apoptosis of alloreactive T cells while sparing proliferating hematopoietic stem cells using preferentially glycolysis [59•]. This strategy was successful in preventing graft vs host disease while preserving immune system repopulation in a mouse model. In a mouse model of systemic lupus erythematosus (SLE), a combination treatment with the glycolysis inhibitor 2-deoxy-d-glucose and the fatty acid oxidation inhibitor metformin was able to normalize bio-energetic metabolism of T cells and reverse SLE [60•]. As previously reported, there is a strong link between IL-7 signaling and bio-energetic metabolism, especially the IL-7 dependent activation of the glycolysis machinery. The T cell bio-energetic metabolism is a novel and quickly developing frontier for developing innovative approaches to control IL-7-mediated homeostatic T cell proliferation and beta-cell autoimmunity.

## Conclusions

IL-7-mediated homeostatic proliferation T cell clones have gained increasing interest in the pathogenesis of type 1 diabetes and in autoimmunity recurrence post pancreas or islet transplantation. This prompted efforts into a better understanding of the molecules and pathways involved in the IL-7/IL-7R axis signaling that can be selectively targeted to control homeostatic proliferation. Some of these are in first phase clinical trials, and we need to consider other ways in which homeostatic proliferation can be controlled and incorporate such strategies in the therapy of beta-cell autoimmunity.
